# First report of *Paratylenchus lepidus* Raski, 1975 associated with green tea (*Camellia sinensis* (L.) Kuntze) in Vietnam

**DOI:** 10.21307/jofnem-2020-110

**Published:** 2020-10-26

**Authors:** Thi Mai Linh Le, Huu Tien Nguyen, Thi Duyen Nguyen, Quang Phap Trinh

**Affiliations:** 1 Institute of Ecology and Biological Resources, Vietnam Academy of Sciences and Technology, 18 Hoang Quoc Viet, Cau Giay, 100000, Hanoi, Vietnam; 2 Graduate University of Science and Technology, Vietnam Academy of Sciences and Technology, 18 Hoang Quoc Viet, Cau Giay, 100000, Hanoi, Vietnam; 3 Nematology Research Unit, Department of Biology, Ghent University, K.L. Ledeganckstraat 35, 9000, Ghent, Belgium

**Keywords:** 28S rDNA, COI mtDNA, DNA barcode, Plant-parasitic nematodes, Taxonomy

## Abstract

The pin nematodes, *Paratylenchus* spp., are relatively small nematodes that can feed on a wide range of host plants. The morphological identification of this nematode is greatly hampered by their small size and variable characters. This study provides the first report of *Paratylenchus lepidus* from Vietnam with a combination of morphological and molecular characterizations. The 28S rDNA phylogenetic tree of the genus and the first *COI* mtDNA barcode of this species are also provided.

The genus *Paratylenchus* (Ciobanu et al., 2003) is commonly known as pin nematodes that are ectoparasites and can be frequently found at high density in perennial plants, hop gardens, orchards, or forest trees (Ghaderi et al., 2016; Ghaderi, 2019). Although sometimes plants infected by *Paratylenchus* species show no specific symptoms, large populations of *Paratylenchus* spp. affect the absorption capacity of roots and the general physiology of plants (Ghaderi, 2019). According to Talavera and Navas (2002), *Paratylenchus* is only considered damaging nematodes at a density higher than 500 nematodes per 100 cm^3^ of soil. However, several studies reported that the population of *Paratylenchus* can increase from a low number to damaging levels within a short time (Faulkner, 1964; Brzeski et al., 1975). The identification of *Paratylenchus* species was mostly based on morphological characterizations (Ghaderi et al., 2016), but morphological variation can be an obstacle to the identification process, which make the molecular approach to become more popular in recent studies of pin nematodes. In Vietnam, 16 *Paratylenchus* species have been reported without molecular data, including *Paratylenchus aculentus*, *P. arculatus*, *P colbrani*, *P corbetti*, *P. costatus*, *P. dianthus*, *P. discocephalus*, *P. elachistus*, *P. epicotylus*, *P. laocaiensis*, *P. minusculus*, *P. nawadus*, *P. pandatus*, *P. perlatus*, *P. serricaudatus*, and *P. similis* (Nguyen and Nguyen, 2000; Nguyen et al., 2004). In this study, we provide the first report of *Paratylenchus lepidus* (Raski, 1975) in Vietnam using the combination of morphological and molecular characterizations.

## Material and methods

Soil and root samples were collected from the rhizosphere of green tea (*Camellia sinensis* (L.) Kuntze) in Vietnam. Nematodes were extracted using the modified Baermann tray method (Whitehead and Hemming, 1965). After that, they were fixed and prepared to make permanent slides following Nguyen et al. (2019a). For morphological characterization, measurements and pictures were taken using Carl Zeiss Axio Lab. A1 light microscope equipped with a Zeiss Axiocam ERc5s digital camera. For molecular characterization, the D2-D3 region of 28S rDNA and *COI* mtDNA gene were amplified using D2A/D3B (5′–ACAAGTACCGTGGGGAAAGTTG–3′/5′–TCGGAAGGAACCAGCTACTA–3′) (Subbotin et al., 2006) and JB3/JB4 (5′-TTTTTTGGGCATCCTGAGGTTTAT-3′/5′-TAAAGAAAGAACATAATGAAAATG-3′) (Nguyen et al., 2019b) primers. Forward and reverse sequences were assembled using Geneious R11 (www.geneious.com). The best fit model was chosen using Mega 7 and phylogenetic analysis was done following Nguyen et al. (2019c).

## Results and discussion

### Measurements

*n* = 20 (♀♀): *L* = 340 ± 20 (307-371) µm, *a* = 25 ± 1 (22-27), *b* = 4.1 ± 0.3 (3.7-4.6), *c* = 11.5 ± 1.4 (9.8-13.8), *c*′ = 3.5 ± 0.4 (3.0-4.1), V% = 82 ± 1 (81-84), Lip height = 2.7 ± 0.5 (1.9-3.6) µm, Lip width = 4.9 ± 0.5 (4.2-5.9) µm, Stylet = 25 ± 1 (24-27) µm, Median bulb length = 15.2 ± 2 (12.8-18.0) µm, Median bulb width = 6.9 ± 0.6 (5.9-7.7) µm, SE pore = 75 ± 4 (67-81) µm, Pharynx = 84 ± 3 (79-91) µm, Body width = 13.8 ± 0.4 (13.3-14.5) µm, Vulval body diam. = 12.2 ± 0.4 (11.3-12.6) µm, Anal body diam. = 8.5 ± 0.4 (7.9-9.5) µm, Tail length = 30 ± 3 (26-35) µm.

### Morphological characterization

**Figure 1:**
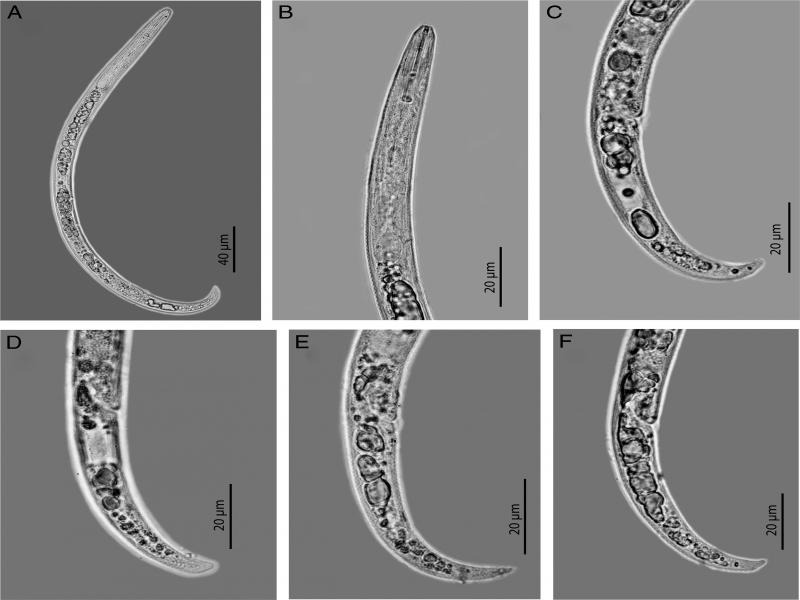
Female of *P. lepidus* from green tea in Vietnam. A: Entire body; B: Anterior end region; C to F: Variation of the tail region.

The female of Vietnamese population of *Paratylenchus lepidus* is characterized by having a slender body, curved ventrally; lateral field with four incisures; lip region weakly sclerotized, continuous to body contour; median bulb elongate with a distinct valve; isthmus slender, surrounded by nerve ring; basal bulb pyriform; secretory-excretory pore located at level of basal bulb to pharyngo-intestinal junction; hemizonid located just anterior to secretory-excretory pore; gonad monodelphic, post uterine sac absent; vulval lips not protruding but having prominent advulval ﬂap; tail curved ventrally with a finely rounded to bluntly pointed terminus (Fig. 1). Male was not found.

### Molecular characterization

**Figure 2:**
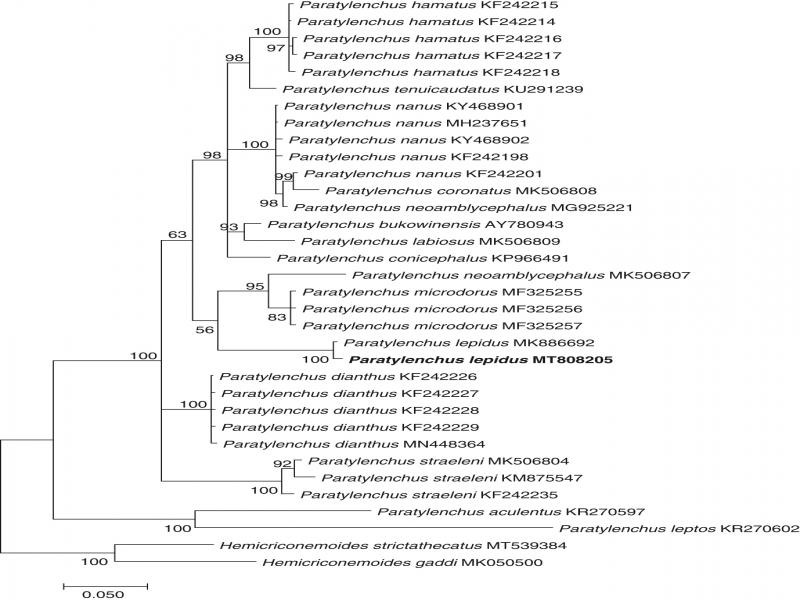
Bayesian inference phylogenetic tree generated from 28S rDNA sequences under HKY + G model (BIC = 2089.633, ln*L* = −740.031, *G* = 0.23, *R* = 3.22, *f*(*A*) = 0.226, *f*(*T*) = 0.182, *f*(*C*) = 0.249, *f*(*G*) = 0.343). Bayesian posterior probabilities (in percentage) are given next to each node. Sequences of *P. lepidus* from Vietnam are in bold font.

The 28S rDNA sequence of *P. lepidus* from Vietnam (742 bp long, accession number: MT808205) was 99.7% similar (2 bp difference) to the sequence of *P. lepidus* from GenBank (accession number: MK886692). The phylogenetic tree based on 28S rDNA sequences showed that the sequence of *P. lepidus* from Vietnam was placed together with the sequence of *P. lepidus* from GenBank (100% PP) (Fig. 2). A *COI* mtDNA sequence of *P. lepidus* from Vietnam (418 bp long) was also obtained and submitted to GenBank under the accession number MT828831.

### Remarks

Morphology of *P. lepidus* from green tea in Vietnam is in agreement with the description of the type population (Raski, 1975) with small variations in measurements, however, these variations can be seen from the type population and other populations (Maria et al., 2019). In this study, molecular identification is in agreement with morphological identification to support the presence of *P. lepidus* in Vietnam. The first *COI* mtDNA sequence of *P. lepidus* is also provided to serve as a molecular barcode for molecular identification of *Paratylenchus* species in the future.
